# Proteomic Mapping of Multifunctional Complexes Within Triatomine Saliva

**DOI:** 10.3389/fcimb.2020.00459

**Published:** 2020-09-02

**Authors:** Paula Beatriz Santiago, Sébastien Charneau, Samuel Coelho Mandacaru, Kaio Luís da Silva Bentes, Izabela Marques Dourado Bastos, Marcelo Valle de Sousa, Carlos André O. Ricart, Carla Nunes de Araújo, Jaime Martins Santana

**Affiliations:** ^1^Pathogen-Host Interface Laboratory, Department of Cell Biology, The University of Brasilia, Brasilia, Brazil; ^2^Laboratory of Protein Chemistry and Biochemistry, Department of Cell Biology, The University of Brasilia, Brasilia, Brazil

**Keywords:** Chagas disease, triatominae, salivary proteins, salivary complexes, BN-PAGE, mass spectrometry

## Abstract

Triatomines are hematophagous insects that transmit *Trypanosoma cruzi*, the etiological agent of Chagas disease. This neglected tropical disease represents a global health issue as it is spreading worldwide. The saliva of Triatominae contains miscellaneous proteins crucial for blood feeding acquisition, counteracting host's hemostasis while performing vasodilatory, anti-platelet and anti-coagulant activities, besides modulating inflammation and immune responses. Since a set of biological processes are mediated by protein complexes, here, the sialocomplexomes (salivary protein complexes) of five species of Triatominae were studied to explore the protein-protein interaction networks. Salivary multiprotein complexes from *Triatoma infestans, Triatoma dimidiata, Dipetalogaster maxima, Rhodnius prolixus*, and *Rhodnius neglectus* were investigated by Blue-Native- polyacrylamide gel electrophoresis coupled with liquid chromatography tandem mass spectrometry. More than 70 protein groups, uncovering the landscape of the Triatominae salivary interactome, were revealed. Triabin, actin, thioredoxin peroxidase and an uncharacterized protein were identified in sialocomplexes of the five species, while hexamerin, heat shock protein and histone were identified in sialocomplexes of four species. Salivary proteins related to triatomine immunity as well as those required during blood feeding process such as apyrases, antigen 5, procalins, and nitrophorins compose different complexes. Furthermore, unique proteins for each triatomine species were revealed. This study represents the first Triatominae sialocomplexome reference to date and shows that the approach used is a reliable tool for the analysis of Triatominae salivary proteins assembled into complexes.

## Introduction

Triatominae (Hemiptera: Reduviidae) are the insect vectors of the flagellated protozoan *Trypanosoma cruzi*, which is the pathogen causative of Chagas disease. According to the WHO, Chagas disease is a neglected tropical disease that affects an estimated 6–7 million people worldwide (WHO, 2020). Originally endemic in Latin America countries, in recent decades the disease showed changes in its epidemiological profile due to migratory events, emerging as a global health concern (Pérez-Molina and Molina, 2018). In non-endemic regions, the main routes of *T. cruzi* transmission are blood transfusion, organ transplantation, and congenital, while the vectorial form occurs in endemic areas where it overlaps with the distribution of triatomine insects (Antinori et al., 2017; Lidani et al., 2019).

The Triatominae subfamily encompasses more than 150 species, grouped in 16 genera and 5 tribes (Monteiro et al., 2018), living in sylvatic, peridomestic, and domestic habitats. After taking a successful blood meal on vertebrates, triatomines are able to transmit *T. cruzi*. In particular, *Triatoma, Rhodnius*, and *Panstrongylus* are genera of epidemiological importance, since some of their species complete the biological cycle in human dwellings. During feeding, hematophagous arthropods are challenged by the host's complex hemostatic and immune responses, which prevent blood loss following bite injury. Triatomines use the salivary compounds to overcome these barriers in order to achieve a continuous blood flow at the bite site (Francischetti et al., 2009). In this scenario, the understanding of vector biology, including the complex interaction of its saliva with the host, is an important research priority.

The transcriptomic and proteomic analyses of Triatominae salivary glands (sialomes) highlight the diversity of putative mechanisms of action of the salivary molecules (Santiago et al., 2020). Indeed, triatomine saliva is an important protein mixture that interacts with the host's molecules performing anti-inflammatory, anti-hemostatic, immunomodulatory, and local anesthetic activities (Ribeiro, 1995). The sialomes provide a deep insight into the biology of these vectors, the important aspects of the blood feeding process, as well as the vector-host interactions (Santiago et al., 2020). Among the salivary proteins, one key enzyme in inhibition of platelet aggregation is apyrase (E.C. 3.6.1.5), already reported in the saliva of several hematophagous organisms including arthropods and leeches (Ware and Luck, 2017). Upon injury to a vessel wall, at the insect bite site, damaged cells and dense granules of activated platelets release adenosine diphosphate (ADP), an important physiological agonist of platelet aggregation. This nucleotide binds to platelet membrane purinergic receptors leading to cell shape change and release of granular contents, triggering a positive feedback mechanism that potentiates platelet activation and aggregation (Kahner et al., 2006). Earlier, we have shown that *Triatoma infestans* apyrases are coordinated in a multiprotein complex with the ability to hydrolyze the agonist ADP, as well as adenosine triphosphate (ATP), into adenosine monophosphate (AMP) and inorganic phosphate (iP) (Faudry et al., 2004a, 2006). Thus, apyrases inhibit ADP-mediated activation of host platelets and subsequent aggregation of thrombocytes to facilitate blood feeding. In addition, the enzyme has also been implicated in modulating host inflammation by ATP degradation (Gounaris and Selkirk, 2005).

A fascinating feature concerning proteins is their ability to form supramolecular structures. In this regard, while exerting their functions, a number of proteins do not work as isolated molecules but rather as part of larger complexes formed by two or more associating polypeptide chains, which may be self- or cross-association, forming, respectively, the homo- and the hetero-oligomers (Ali and Imperiali, 2005). The assembly of subunits is based on the chemical and geometrical complementarities and can be either transient or stable (Nooren and Thornton, 2003). Homomers together with heteromers make up more than 50% of protein stoichiometry distribution in Protein Data Bank (PDB, 2020). Functional roles of oligomeric states might be crucial for the regulation of a series of biological processes, mediating gene expression and enzyme activity.

Among a large range of applications, native polyacrylamide gel electrophoresis (BN-PAGE) is a useful approach to analyze native oligomeric states in protein complexes (Schägger et al., 1994; Wittig et al., 2006; Miernyk and Thelen, 2008). Complexes migrate as blue bands through BN-PAGE gels, so they can be sliced off and be subjected to mass spectrometry analyzes, identifying their multiprotein composition. This combined approach provides size and protein-protein interaction data. Moreover, to identify oligomeric states activity, native complexes can be analyzed by in-gel activity assay.

The objective of this work was to unveil the protein-protein interaction network in the saliva (sialocomplexome) of *T. infestans, Triatoma dimidiata, Dipetalogaster maxima, Rhodnius prolixus*, and *Rhodnius neglectus*. Therefore, we separated and identified salivary multiprotein complexes by blue-native PAGE following mass spectrometry analysis. In addition, the oligomeric apyrase enzyme activity was also investigated by zymographic BN-PAGE. To our knowledge, this is the first insight into the sialocomplexomes of Triatominae.

## Materials and Methods

### Triatomines and Saliva Collection

Triatomine bugs were reared in the insectarium of the University of Brasília (Brasília, Brazil). They were kept at 27 ± 1°C, a relative humidity of 70–75%, under a 12 h/12 h light/dark cycle. The blood source of these insects was *Gallus gallus domesticus*. Twenty-one days post-feeding, as salivary content 20 days after feeding is representative (Faudry et al., 2004a), 15 adults from both sexes of *T. infestans, T. dimidiata, D. maxima, R. prolixus*, and *R. neglectus*, were immobilized on ice, and under a stereomicroscope the salivary content was obtained by dissection of their salivary glands (SG), which were carefully washed in sterile saline solution before being harvested in microtubes containing EDTA-free protease inhibitor cocktail (Roche, Switzerland), and placed on ice. The pools of saliva from each species were centrifuged at 16,000 × g for 15 min at 4°C. The supernatant collected was kept at −80°C prior to use. The protein concentrations were determined using the Qubit® Protein Assay Kit (Thermo Fisher Scientific).

### BN-Page

The salivary gland homogenates of different triatomines were subject to 1D BN-PAGE, as previously described for soluble proteins (Schägger et al., 1994; Wittig et al., 2006). The crude salivary extract from each species (150 μg of total protein) was resuspended in a sample buffer (50 mM Bis-Tris HCl pH 7.0, 15% (w/v) glycerol), and the samples were loaded onto a polyacrylamide gradient gel of 5–18%. Electrophoresis was run at 15 mA constant current for 4 h, at 4°C, in a SE 600 electrophoresis system (Hoefer, Inc., San Francisco, CA, USA). The cathodic buffer consisted of 15 mM Bis-Tris HCl pH 7.0, 50 mM Tricine and 0.02% (w/v) Coomassie Brilliant Blue G-250 (CBB G-250), while the anodic buffer was composed of 50 mM Bis-Tris HCl pH 7.0. Once electrophoresis was complete, gels were either fixed and stained using CBB G-250 or used for the zymograms. Using a scalpel, the blue bands with high staining intensity containing the potential protein complexes were cut from the gel and stored at −20°C for subsequent LC-MS/MS analysis. The proteins thyroglobulin (669 kDa), ferritin (440 kDa), catalase (232 kDa), aldolase (158 kDa), bovine serum albumin (67 kDa), and ovalbumin (43 kDa) were used as molecular weight markers.

### LC-MS/MS Protein Identification

Gel slices excised from the 1D-BN-PAGE were prepared for LC-MS/MS analysis by washing twice 50% acetonitrile por 4 h (in order to remove CBB-250), dehydrating in 100 μL of acetonitrile (ACN) and drying in a speed vacuum (Eppendorf, Hamburg, Germany). The proteins were in-gel reduced with 20 mM dithiothreitol in 25 mM ammonium bicarbonate buffer at 56°C for 45 min and alkylated with 40 mM iodoacetamide in the same buffer at room temperature, in the dark, for 1 h. Subsequently, in-gel proteins were washed in ACN and digested overnight at 37°C with 12.5 ng/μL modified trypsin (Promega, Madison, USA). The supernatants were acidified to a final concentration of 0.1% trifluoroacetic acid (TFA) for the first tryptic peptide extraction and followed by two other steps consisting in 0.1% TFA (v/v) in 50% ACN (v/v) and 0.1% TFA (v/v) in 80% ACN (v/v), respectively. The samples were then lyophilized, solubilized in 50 μL 1% TFA and desalted on a pipette tip packed with C18 membrane (Empore, Supelco). The digests were washed 3 times with 0.5% acetic acid and the peptides eluted with 25, 50, 80, and 100% ACN solutions, all containing 0.5% acetic acid (v/v), prior to LC-MS/MS analysis using an Orbitrap Elite^TM^ hybrid ion trap- orbitrap mass spectrometer (Thermo Fisher Scientific Inc., Waltham, MA, USA). After 4 min washing with solvent A (0.1% (v/v) formic acid), the samples resuspended in 0.1% (v/v) formic acid were loaded into a nano-UPLC-Dionex 3000 system (Thermo Fisher Scientific Inc., Waltham, MA, USA) equipped with a trap type C18 column (100 μm × 3 cm with particles of 5 μm/100 Å) and a C18 analytical column (75 μm × 35 cm with particles of 3 μm/100 Å). The peptides were eluted from the analytical column at a flow rate of 230 nL.min^−1^ directly into the mass spectrometer under ESI ionization with a gradient of 2 to 35% of solvent B (0.1% [v/v] formic acid in ACN) for 30 min, 35–90% B and 90% B for 10 min, and decrease to 2% B to equilibrate the column for 20 min. The Data Dependent Acquisition (DDA) cycle of acquired molecular mass spectra controlled by Xcalibur 2.0 software (Thermo Fisher Scientific Inc., Waltham, MA, USA) comprised the range of m/z 350 to 1,650 and resolution of 120,000 for MS1. The fifteen most abundant precursor ions were fragmented by high energy collision dissociation (HCD), when the MS2 detection was performed at 60,000 resolution in the orbitrap analyzer, with dynamic exclusion for 90 s and collision energy normalized to 35%.

### Data Analysis

Raw files were generated by the spectrometer and imported by PEAKS Studio 7.0 (Bioinformatics Solution Inc., Waterloo, Canada) software. Mass spectra data from samples were searched against the Uniprot Triatominae database (57,736 sequences accessed on 04/02/2020) and protein sequences of known contaminant proteins (several human keratins, BSA, and porcine trypsin). The parameters used were 10 ppm peptide mass tolerance, 0.5 Da fragment mass tolerance and two missed cleavages allowed. Methionine oxidations and acetylation of protein N-termini were specified as variable modifications, while carbamidomethylation of cysteine was specified as a fixed modification. Positive protein identities were assigned if at least one unique peptide were matched using a false discovery rate (FDR) of <1%. All contaminant proteins identified were manually removed from the result lists.

As a result of the redundancy of protein databank and protein isoforms derived from the same gene, over-counting of protein inferences might occur. Here, we used a clustering method where an identified protein is classified in a specific protein group (PG) when it has the assignment corresponding to a particular protein family. To be more consistent, these results were subsequently manually curated.

### Data Availability

Mass spectrometer output files (Raw data) are available from the MassIVE database (accession number MSV000085118, doi: 10.25345/C5WT30, (http://massive.ucsd.edu/ProteoSAFe/status.jsp?task=364af77ff94b42729ffa5dcaf49fecd0) and ProteomeXchange (accession number PXD018101) (Vizcaíno et al., 2014; Perez-Riverol et al., 2015; Jarnuczak and Vizcaíno, 2017).

### Zymogram

In-gel apyrase activity was based on the formation of a white insoluble calcium phosphate precipitate following phosphate production by ADP hydrolysis (Valenzuela et al., 1998). *T. infestans, T. dimidiata, D. maxima, R. prolixus*, and *R. neglectus* salivary contents (150 μg) were subjected to native 1D-BN-PAGE at 4°C. Immediately after running, the gel was washed twice with cold 2.5% (v/v) Triton X-100 and cold Milli-Q water, alternatively for 20 min each wash. The gel was incubated in activity solution (50 mM Tris-HCl, pH 8.3; 100 mM NaCl; 20 mM CaCl_2_; 20 mM MgCl_2_; 5 mM ADP) at 37°C for 30 min. The reaction was stopped with the same solution in the absence of the nucleotide.

## Results

### Triatominae Salivary Protein Complexes Exhibit Unique Profiles

**Figure 1:**
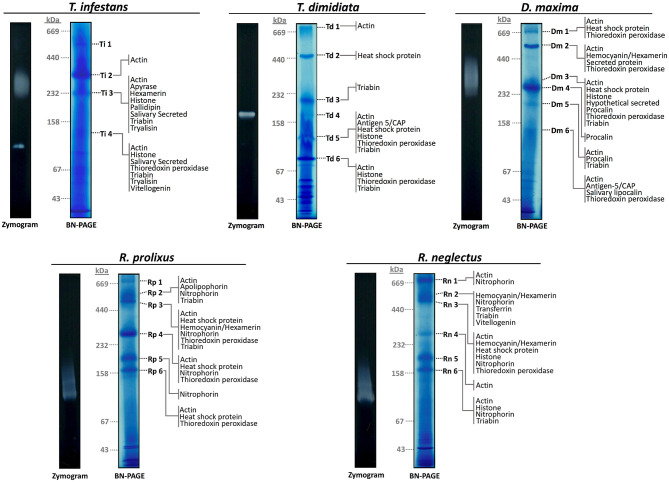
Sialocomplexomes profile and ADPase activity from *Rhodnius prolixus, Rhodnius neglectus, Dipetalogaster maxima, Triatoma dimidiata*, and *Triatoma infestans* in 1D BN-PAGE. Protein complexes present in the saliva from *R. prolixus, R. neglectus, D. maxima*, and *T. dimidiata* are numbered from 1 to 6, and from *T. infestans* are numbered from 1 to 4. Zymography/BN-PAGE assays evaluating the apyrase activity are given to the left of the gels. Gels were run according to the protocols given in Methods. The positions of gel slices are shown by the species initials and number of the band. Hypothetically secreted proteins identified in each band are presented. Gels gradient were 5–18% and they were stained with CBB G-250. Molecular masses (in kDa) are given to the left of the Coomassie-stained gels.

1D-BN-PAGE allowed the separation of the potential salivary multiprotein complexes (Figure 1). To minimize technical variation, samples from the five different species were processed in parallel. *R. prolixus* and *R. neglectus* 1D-BN-PAGE maps were almost identical whereas *T. infestans, D. maxima*, and *T. dimidiata* displayed unique profiles. Each 1D-BN-PAGE band above 67 kDa was considered as a potential multiprotein complex. In all five species, it is possible to observe that some protein complexes ran above 440 kDa molecular weight. Four potential heteromeric protein complexes were obtained from salivary extracts of *T. infestans* (Ti-1-4), and six from *T. dimidiata* (Td 1-6), *D. maxima* (Dm1-6), *R. prolixus* (Rp1-6), and *R. neglectus* (Rn 1-6) (Figure 1).

### The Composition of Triatominae Sialocomplexomes

**Figure 2:**
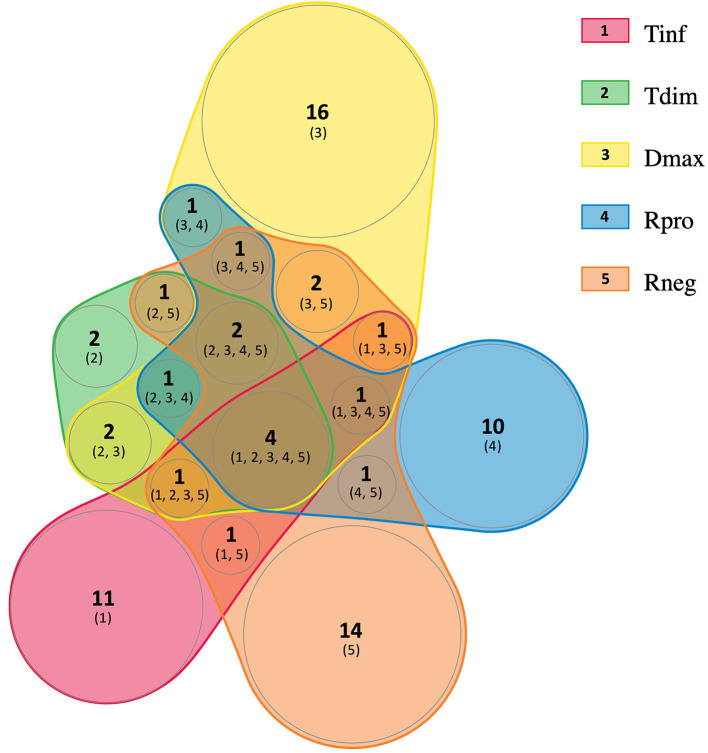
Venn diagram analysis of the protein groups identified in Triatominae sialocomplexomes. Tinf, *Triatoma infestans*; Tdim, *Triatoma dimidiata*; Dmax, *Dipetalogaster maxima*; Rpro, *Rhodnius prolixus*; Rneg, *Rhodnius neglectus*. The diagram was created using nVenn toll (Pérez-Silva et al., 2018).

To determine the subunit composition in the protein complexes, the four potential multiprotein bands with high staining intensity from *T. infestans*, and the six from *T. dimidiata, D. maxima, R. prolixus*, and *R. neglectus* were individually submitted to LC-MS/MS analysis. A comprehensive list of all identified proteins and peptides is provided in Supplementary Files 1, 2, respectively. A total of 72 protein groups (PGs) were identified and several PGs were present in different bands from different triatomine saliva. It was possible to identify 19 different PGs in *T. infestans* sialocomplexomes, while 13 and 32 PGs were identified in *T. dimidiata* and *D. maxima*. *R. prolixus* and *R. neglectus* displayed 21 and 29 PGs, respectively. Figure 2 shows a diagram representing the relationships among the sets of PGs identified on sialocomplexomes from the five species studied.

The sets of PGs were classified into five categories based on their putative biological functions as follows: Hypothetically secreted, Enzyme, Housekeeping, Unknown, and *Rhodococcus rhodnii-*derived proteins, condensed in Table 1. Remarkably, 24 (33.33%) of the PGs were classified as Hypothetically secreted, which are composed by proteins associated with hematophagy. The Enzyme class had 12 (16.67%) of all PGs. The Housekeeping class had 23 PGs, corresponding to 31.94% of the total identified. Approximately 11.11%, corresponding to 8 PGs, were not classified in the Triatominae Uniprot database and were placed in the Unknown class. *Rhodococcus rhodnii-*derived proteins accounted for 6.94% of the PGs.

**Table 1:** Functional classification of the Protein Groups (PGs) identified on Triatominae sialocomplexomes.

**Class**	**Number of PG**	**% PGs**
Hypothetically secreted	24	33.33
Enzyme	12	16.67
Housekeeping	23	31.94
Unknown	8	11.11
*Rhodococcus rhodnii*-derived products	5	6.94

The proteins identified in each BN-PAGE complex band can be seen in Table 2 and in Figure 1. Four PGs were common to all five species, three in the Hypothetical secreted class, which are: (i) actin, (ii) thioredoxin peroxidase, (iii) triabin; and (iv) an uncharacterized protein (T1HZ69) from the Unknown class. The hemocyanin/hexamerin, the heat shock protein and the histone PGs were identified in the majority of the species, except for *T. dimidiata, T. infestans*, and *R. prolixus*, respectively.

**Table 2:** List of Triatominae salivary proteins identified on the oligomeric protein complexes and their putative functions.

**Protein group^**a**^**	**Putative molecular function^**b**^**	**BN-PAGE band^**c**^**
**HYPOTHETICALLY SECRETED**		
Apyrase		
79 kDa salivary apyrase	Hydrolysis of ADP and ATP	Ti 3
Salivary apyrase	Hydrolysis of ADP and ATP	Ti 3
Actin	Insect immunity/Inhibition of platelet aggregation	Ti 2; Ti 3; Ti 4; Td 1; Td 5; Td 6; Dm 1; Dm 2; Dm 3; Dm 5; Dm 6; Rp 2; Rp 3; Rp 4; Rp 6; Rp 7; Rn 1; Rn3; Rn 4; Rn 6
Antigen-5/CAP family		
Antigen-5-like protein	Inhibition of platelet aggregation; Inhibition of coagulation; Antioxidant activity	Dm 6
SCP domain-containing protein	Inhibition of platelet aggregation; Inhibition of coagulation; Antioxidant activity	Td 5
Cytochrome P450-like protein	Heme binding	Td 6
Histone		
Histone H2A	Insect immunity	Ti 4; Td 5; Td 6; Rn 3
Histone H2B	Insect immunity	Ti 3; Rn 3
Histone H3	Insect immunity	Ti 4; Td 6; Dm 3; Rn3; Rn 6
Hypothetical secreted protein (E2J7B4)	Unknown	Dm 3
Ig-like domain-containing protein	Unknown	Ti 3
Hemocyanin/Hexamerin		
Larval serum protein 2	Insect immunity	Ti 3; Dm 2
Putative basic juvenile hormone	Insect immunity	Dm 2; Rn 2
Putative hexamerin	Insect immunity	Ti 3; Dm 2
Uncharacterized protein (T1HU08)	Insect immunity	Dm 2; Rp 3; Rn2; Rn 3
Nitrophorin		
Nitrophorin 1	Inhibition of platelet aggregation; Vasodilatory; Anti-histaminic	Rp 2; Rp 3; Rp 4; Rp 7; Rn2; Rn3
Nitrophorin 4A	Inhibition of platelet aggregation; Vasodilatory; Anti-histaminic	Rn 2
Nitrophorin-3	Inhibition of platelet aggregation; Vasodilatory; Anti-histaminic	Rn 2; Rp 5
Putative nitrophorin	Inhibition of platelet aggregation; Vasodilatory; Anti-histaminic	Rp 2; Rp 3; Rn1; Rn2; Rn3
Small nitrophorin 2A	Inhibition of platelet aggregation; Vasodilatory; Anti-histaminic	Rp 7; Rn 6
Pallidipin-like salivary lipocalin	Inhibition of platelet aggregation	Ti 3
Procalin	Salivary allergen	Dm3; Dm4; Dm5
Putative apolipophorins	Lipid transporter	Rp 2
Putative drim down-regulated in metastasis-like	Heme binding	Rn 2
Putative heat shock protein	Protein folding, Fibrinogenolysis	Td 2; Td 5; Dm1; Dm3; Rp 3; Rp 4; Rp 6; Rn3
Putative pdz domain-containing protein	Protein-protein interactions mediator	Rp 4
Putative salivary secreted protein	Actin-crosslinking	Ti 4
Putative secreted protein	Unknown	Dm 2
Putative thioredoxin peroxidase	Detoxification of ROS	Ti 4; Td 5; Td 6; Dm 1; Dm 2; Dm 3; Dm 6; Rp 3; Rp 4; Rp 6; Rp 7; Rn3
Salivary lipocalin	Inhibition of hemostasis	Dm 6
Salivary secreted protein	Unknown	Ti 3
Transferrin	Insect immunity	Rn 2
Triabin	Inhibition of thrombin	Ti 3; Ti 4; Td 3; Td 5; Td 6; Dm 3; Dm 5; Rp 2; Rp 3; Rp 7; Rn2; Rn 6
Trialysin	Pore-forming lytic protein	Ti 3; Ti 4
Vitellogenin lipoprotein	Lipid transporter	Ti 4; Rn 2
**ENZYME**		
Aminopeptidase	Peptidase M1/MetalloAminopeptidase activity	Dm 2
Fanconi-associated nuclease	Phosphodiesterase I activity	Rp 4
Peptidylprolyl isomerase	Peptidyl-prolyl cis-trans isomerase activity	Td 6; Dm 2; Rp 5
PlsC domain-containing protein	Transferase activity, transferring acyl groups	Rp 1
Putative acetyl-coa carboxylase biotin carboxylase	Biotin carboxylation	Rn 2
Putative heparan sulfate-glucuronic acid c5-epimerase	Heparosan-N-sulfate-glucuronate 5-epimerase activity	Dm 2
Putative trna modification enzyme	tRNA [guanine(37)-N(1)]-methyltransferase activity	Td 6; Rn 2; Rn 6
Putative trypsin-like serine protease	Serine-type endopeptidase activity	Ti 4
Putative vacuolar h+-atpase v1 sector subunit e	Proton-transporting two-sector ATPase	Dm 2; Rp 6
Serine/threonine-protein kinase	Protein serine/threonine kinase activity	Rn 2
Uncharacterized protein (T1HWH5)	Peptidase_M16/metalloendopeptidase activity	Dm 2
Uncharacterized protein (T1I287)	Peptidase_M13/metalloendopeptidase activity	Rn 2
**HOUSEKEEPING**		
Circadian trip	Ubiquitin-protein transferase activity	Dm 4
CXXC-type domain-containing protein	DNA-binding	Rn 6
Dynein	Microtubule motor activity	Rn 2; Rn 6
Elongation factor 1-alpha	Translation elongation factor activity	Td 5; Td 6
Putative cell cycle control protein crooked neck	RNA processing	Rn 2
Putative endoplasmic reticulum glucose-regulated	ATP binding/protein folding	Dm 1
Putative golgin subfamily protein a member	Golgi organization	Dm 2
Putative guanine nucleotide binding protein mip1	TOR signaling	Ti 3
Putative mediator of rna polymerase ii	Transcription coregulator activity	Dm 2; Dm 3
Putative muscle-specific protein	Cytoskeletal structure	Rn 6
Putative myosin class ii heavy chain	Cytoskeletal structure	Rp 3
Putative retrotransposon-like family	Nucleic acid binding	Dm 3
Putative ribosome-binding protein 1-like isoform x5	Ribosome-binding protein	Ti 4
Putative titin isoform x14	Immunoglobulin-like domain	Rp 4
Putative transcription regulator xnp/atrx dead-box	Nucleic acid binding	Rn 2
Putative ubiquitin	Protein regulation	Ti 3; Dm 2; Rn 2; Rn 3
Putative unconventional myosin-xviiia	Cytoskeletal structure	Td 6; Dm 3
Retinoid-and fatty acid-binding glycoprotein	von Willebrand factor type D domain	Ti 2
Tropomyosin isoform	Cytoskeletal structure	Dm 2; Rp 4; Rn 3
Uncharacterized protein (T1HAV7)	RNA binding/processing	Rn 6
Uncharacterized protein (T1HA71)	DNA binding/regulation of transcription	Rp 7
Uncharacterized protein (T1I899)	DNA binding	Dm 2
Uncharacterized protein (T1IAP4)	Integral component of membrane	Dm 3
**UNKNOWN**		
Uncharacterized protein (T1HZ69)	-	Ti 4; Td 6; Dm 1; Dm5; Rp1; Rp 3; Rp 4; Rn1
Uncharacterized protein (T1HNL8)	-	Dm 2
Uncharacterized protein (T1HS38)	-	Dm 2; Rn 3
Uncharacterized protein (T1HK09)	-	Rn 6
Uncharacterized protein (T1HX12)	-	Rp 5
Uncharacterized protein (A0A069DS40)	-	Dm 2; Rn 3
Uncharacterized protein (T1HU42)	-	Rp 4
Uncharacterized protein (T1IDW9)	-	Rp 1
***RHODOCOCCUS RHODNII***		
Elongation factor	Translation elongation factor activity	Rn 6
HNHc domain-containing protein	-	Td 6; Dm 1; Rp 2; Rn 1; Rn 5; Rn 6
P-type ATPase	-	Dm 2
Transferase	Transferase activity	Ti 3
Uncharacterized protein (R7WS93)	-	Rn 6

a *Protein group name based on protein signature identified by LC/MS-MS*.

b *For the Hypothetical secreted PGs: Putative function of the particular protein group in blood feeding context (vector/host interaction). See references in discussion section. For the Housekeeping and R. rhodnii PGs: Molecular function were based on their classification at Uniprot database*.

c *Triatomine species initials and number of the BN gel band submitted to LC/MS-MS identification*.

Regarding the Hypothetically secreted class, with the exception of *T. dimidiata*, the species exhibited unique PGs. Apyrase, Ig-like domain-containing protein, pallidipin-like salivary lipocalin, putative salivary secreted protein, and salivary secreted protein PGs were identified only in *T. infestans*; the hypothetically secreted protein, procalin, and putative secreted protein PGs in *D. maxima*; putative apolipophorin, and putative pdz domain-containing protein PGs in *R. prolixus*; and putative drim down-regulated in metastasis-like and transferrin PGs in *R. neglectus*. Furthermore, *Rhodnius* genera showed the unique nitrophorin PG.

### Sialocomplexomes From Triatominae Present ADPase Activity in Gel

The apyrase activity was detected above 232 kDa, and in a single band below 158 kDa in *T. infestans*; between 158 and 232 kDa in *T. dimidiata;* and above 232 kDa in *D. maxima*. A smear activity below 158 kDa was observed in both *R. prolixus* and *R. neglectus* (Figure 1). The activity smear/bands revealed in *T. infestans, T. dimidiata*, and *D. maxima* merged with sialocomplexes Ti 3, Td 4, and Dm 3. While enzymatic activity profiles of *R. prolixus* and *R. neglectus* were not associated with stained-CBB bands.

## Discussion

### Classes of Proteins in the Triatominae Sialocomplexomes

Multiprotein complexes are macromolecular assemblies that regulate essential cellular and physiological processes within biological systems. Despite progresses having been made regarding knowledge of Triatominae saliva and its fundamental role in blood acquisition during feeding (Santiago et al., 2020), insights about the composition and interaction network of salivary protein complexes from triatomines remain unavailable. The proteomic approach used here revealed the salivary proteins identified on BN-PAGE bands ran above their predicted molecular weights, suggesting they form potential protein complexes of higher-order assemblies. Here, when taking a closer look into *Rhodnius* species, a similar electrophoretic profile on BN-PAGE maps from *R. prolixus* and *R. neglectus* was evident. Conversely, Triatomini species showed a diversification of their salivary electrophoretic profiles, in agreement with the observation that this tribe lineage diversified extensively and consistent during evolution. Although, species and genera from Rhodniini and Triatomini tribes exhibit a variety of ecological, morphological, and molecular (salivary proteins) characteristics, recent phylogenetic analyses have suggested the monophyly of the Triatominae subfamily (Monteiro et al., 2018).

As detailed in Table 1, around 32% of identified proteins in BN-PAGE maps were attributed to the Housekeeping class. Different proteins associated with DNA or RNA binding/processing, protein synthesis and regulation, cytoskeletal structure were revealed. Previous report suggested a contamination of proteins from salivary gland tissue might have occurred due to salivary glands dissection and saliva collection procedures, not reflecting secreted proteins from saliva (Santiago et al., 2018). However, another hypothesis, supported by the observation that a tick heat-shock cognate protein 70 (HSP70) that interferes with host fibrinogenolysis at the bite site was reported in tick-cell-line-derived exosomes (Vora et al., 2017), is that salivary exosomes may deliver some of these proteins to exert specific biological functions in vertebrate hosts (Hackenberg and Kotsyfakis, 2018). Thus, in our analysis, actin, heat shock protein, histone, and thioredoxin peroxidase, which have already been reported to have a role when secreted, were classified in the Hypothetically secreted class together with typical salivary proteins. New roles for housekeeping secreted proteins may still be unveiled in future works.

Enzymes are abundant in the saliva of plant-feeding hemipterans (Sharma et al., 2014). Here, we showed that enzymes comprised 16% of the proteins identified in the sialocomplexomes. Triatominae subfamily of hemipterans evolved from predatory reduviid bugs, and during the transition to a hematophagous lifestyle, changes in the function of some salivary enzymes may have occurred (Monteiro et al., 2018). Serine and metalloproteases were already reported in different Triatominae sialomes (Assumpcao et al., 2008; Assumpção et al., 2012; Ribeiro et al., 2015; Santiago et al., 2016, 2018), and although their specific roles are unknown, hematophagous salivary enzymes have been associated with host's anti-hemostatic activities during blood acquisition and immunity (reviewed in Santiago et al., 2017). A trypsin-like serine protease, named triapsin, was reported in the salivary glands of *T. infestans* (Amino et al., 2001), and here, was identified in Ti 4 sialocomplex. Triapsin is stored as a zymogen in the luminal content of the SG, and activated during salivation stimulated by biting, suggesting it has a role in blood feeding (Amino et al., 2001). This protease may also be involved in hydrolysis of the superfamily of Proteinase Activated G protein-coupled Receptors (PAR), which regulates growth, development, inflammation, and responses to injury (Amino et al., 2001).

Three metalloproteases were also identified in this study. The aminopeptidase identified in Dm 2 is a member of the M1 family of Zn^2+^ metalloproteases, which are ubiquitous enzymes implicated in many physiological functions (Hooper, 1994). M1 aminopeptidases are found in all insect orders and have been detected in various tissues including salivary glands. They also play important roles in protein digestion (Budatha et al., 2007) and host-pathogen interactions (Denolf et al., 1997; Rajagopal et al., 2003; Aroonkesorn et al., 2015). In Hemipterans, triatomine aminopeptidases have been mainly implicated in the digestion of blood proteins (Garcia et al., 2010). Another zinc-dependent metalloprotease, a member of M16 family (T1HWH5), identified in Dm 2 may also be involved in digestion. In Rn 2, a member of the M13 family of zinc-dependent metallopeptidases was identified. Enzymes from this family have a broad tissue distribution in insects (Macours and Hens, 2004), and have been implicated in metamorphosis (Wilson et al., 2002), immunity to bacteria, fungi and protozoa (Zhu et al., 2003; Aguilar et al., 2005), neuropeptide metabolism (Isaac et al., 2009), and reproduction (Sitnik et al., 2014). Additionally, nuclease, isomerase, carboxylase, transferase, among others (Table 2) were also identified in the triatomine sialocomplexomes. These enzymes act on enzymatic cascades regulating various biological processes. The precise function of enzymes in sialocomplexes is unknown, but transient enzymatic complexes association into higher order assemblies have regulatory roles in biological functions.

In the Uncharacterized class, nine proteins with unknown functions were identified. Among them, only the protein T1HZ69 was ubiquitous in the five triatomine species. Another interesting result was the identification of proteins from *Rhodococcus rhodnii* among the sialocomplexomes from all five species. This bacterium was reported composing the low-diversity microbiota associated with the Triatominae salivary glands and was shown to be fundamental to the biological fitness of *R. prolixus* (Lima et al., 2018). *Rh. rhodnii* are members of the Nocardiaceae family, which are symbiont bacteria from triatomines. They are passed from adults to offspring by coprophagy and appear to supplement, the triatomine diet, especially with B vitamins (Brecher and Wigglesworth, 1944). Five proteins from *Rh. rhodnii* were identified. Among them, a HNHc domain-containing protein was detected not only in *R. prolixus*, but also in *R. neglectus, T. dimidiata* and *D. maxima*. The HNHc domain is associated with DNA-binding proteins involved in cellular processes such as recombination, DNA rearrangement, phage packaging, restriction endonuclease activity and bacterial toxicity (Dalgaard et al., 1997). The possible roles of *Rh. rhodnii* proteins in the saliva of triatomines warrants further investigation.

Our major interest is the Hypothetically secreted proteins since they are involved in the triatomine-host interface. This class accounted for 32% of the identified proteins. When comparing results from the five species, it was seen that actin, thioredoxin peroxidase, triabin, uncharacterized (T1HZ69), hemocyanin/hexamerin, heat shock protein and histone and were identified in various BN-PAGE bands. While the former four are ubiquitous, the last three were not found in *T. dimidiata, T. infestans*, or *R. prolixus*, respectively. Apart from triabin, none of the other six proteins were previously highlighted in triatomine saliva before. It is likely that reports have focused on secreted salivary molecules with putative functions already described in literature. Multifunctional molecules, those that await identification of their salivary function or interact with ligands not yet characterized, remain unnoticed.

Proteins commonly described in Triatominae saliva were also revealed. *Rhodnius* sialocomplexes exhibited interacting nitrophorins, which are nitric oxide (NO)-binding heme proteins with vasodilatory function (Champagne et al., 1995). *T. infestans* saliva is distinguished by the presence of apyrases, pallidipin, and trialysin; *T. dimidiata* by antigen-5/CAP; and *D. maxima* by antigen-5/CAP, and procalin (Table 2). These molecules account for a diversity of molecular mechanisms of triatomine saliva to overcome host's hemostasis. Pallidipin and antigen-5/CAP members are inhibitors of platelet aggregation mediated by collagen (Noeske-Jungblut et al., 1994; Assumpção et al., 2013). Triabin is a thrombin inhibitor (Noeske-Jungblut et al., 1995), while trialysin is pore-forming lytic protein (Amino et al., 2002), and procalin is a salivary allergen (Paddock et al., 2001). Nitrophorin, pallidipin, procalin, triabin, among other proteins identified here, belong to the lipocalin/calicin superfamily of proteins, which is comprised of low molecular weight proteins with mainly extracellular functions. These proteins share a common structure and are able to bind to small hydrophobic molecules, soluble macromolecules, macromolecular complexes, through non-covalent and covalent bonds; and to specific cell surface receptors (Hernández-Vargas et al., 2016).

Previous triatomine sialome analyses revealed this superfamily is the most abundant and functionally diverse from saliva (reviewed in Santiago et al., 2020).

### Salivary Apyrases of Triatominae Are Assembled in Complexes

Apyrase in-gel activity was detected in all samples. In *T. infestans* saliva, an activity band under 158 kDa and a smear above 200 kDa, which is the result of aggregates of active small units, were detected. Moreover, apyrase members were identified by proteomics in Ti 3 sialocomplex, corroborating the in-gel activity is due to true apyrase salivary proteins. This result is in accordance with the 200 kDa apyrase homo-oligomers from the saliva of *T. infestans* reported before (Faudry et al., 2006). A similar pattern of smeared apyrase activity was observed between *T. infestans* and *D. maxima*, and between *R. prolixus* and *R. neglectus*. The various aggregates may reflect several post-translational modifications of the salivary apyrase units, and even other protein units, they contain. Concerning *T. dimidiata*, a single active form (above 158 kDa) was observed. Therefore, different molecular weights were shown to these ADPase activities depending on the triatomine species, indicating heterogeneity of enzyme isoforms and oligomerization.

Although the apyrase in-gel activity was detected in all the samples tested in this study, the specific *T. infestans* apyrase sequences present in the database were not identified by mass spectrometry in *T. dimidiata, D. maxima, R. prolixus*, and *R. neglectus* saliva bands. This result appears to reflect the presence of a small amount of apyrase proteins in saliva. In addition, considering the knowledge about the function and composition of the saliva from triatomines, this hydrolase activity acting on ADP typically occurs due to true apyrase (ATP diphosphohydrolase, EC 3.6.1.5) enzymes (Sarkis et al., 1986; Ribeiro, 1995), corroborating this enzyme is well-conserved among triatomines. Because apyrase efficiently affects platelet aggregation, this enzyme has potential as pharmacological candidate for the treatment of thrombotic pathologies, reflecting its importance for research purposes.

### Multifunctional Proteins in the Triatominae Sialocomplexomes

Actin is one of the most conserved gene families in Eukarya. Isoforms are expressed in different tissues and are involved in a variety of biological processes, mostly intracellular such as muscle contraction, cell motility, cell division, vesicle and movement (Pollard, 2016). Actin monomers spontaneously polymerize with themselves under physiological conditions (Pollard, 2016), and here may form some homo-oligomeric complexes. Furthermore, in the presence of divalent cations, actin monomers tightly bind adenosine triphosphate (ATP) or diphosphate (ADP) (Pollard, 2016), and thus, polymerized secreted actin may have a role in the counteraction of host's hemostasis by binding to these nucleotides. Despite the description of extracellular actins is not common in insects, it has been proposed that actin is externalized by insect immune competent cells upon immune challenge, mediating antibacterial defense (Sandiford et al., 2015). Actin 5C is a high affinity bacterial binding protein (Sandiford et al., 2015). Here, different actin 5C proteins were identified in salivary complexes by proteomic analyses. These proteins are constitutively expressed and as well as composing the cytoskeletal structure, non-standard functions for triatomine salivary actin can be proposed. In this context, actin 5C proteins may have a role in hematophagy, inhibiting platelet aggregation or in immunity, inhibiting the growth of bacteria. This is the first-time that secreted actin proteins are mentioned to play a role in triatomine saliva.

Arthropod hemocyanins (Hc) are large and multimeric members of a protein superfamily that also comprises the arthropod phenoloxidase (PO), crustacean pseudohemocyanins, and insect storage hexamerins (Burmester, 2001). Hc participates in microbicidal activity and provides a first line of defense mechanism against microorganisms, emerging as an evolutionary mode of immune surveillance (Coates and Nairn, 2014). Hc and PO show different structures but share almost identical active site architecture, and both contribute to sclerotization of the arthropod cuticle (Decker and Rimke, 1998; Decker et al., 2007). The presence of Hcs in the saliva of triatomines may be related to protection against pathogens acquired during the blood feeding as well as to the initial steps of digestion inside the midgut, once swallowed with the blood meal. Different members from Hc family were found in the sialocomplexomes of *T. infestans, D. maxima, R. prolixus*, and *R. neglecus*. These large extracellular proteins are found as multiples of 2–8 hexamers (Jaenicke and Decker, 2003). It is likely Hcs form large complexes in the triatomine saliva.

Histones are largely known to control chromatin architecture. It has been suggested that a combination of post-translational modification is able to alter histone functions (Ouvry-Patat and Schey, 2007), including histone antimicrobial activity reported in a wide range of organisms (Patat et al., 2004). Here, histone members were observed in *T. infestans, T. dimidiata, D. maxima*, and *R. neglectus*. It is feasible that they have a role in innate immunity of triatomines, for instance, by controlling the population growth of bacteria acquired with blood meal in the salivary glands and midgut.

Reactive oxygen species (ROS), including superoxide anions (O^2−^), hydroxyl radicals (OH^−^), and hydrogen peroxide (H_2_O_2_), are reactive molecules generated as by-products of aerobic metabolism. High levels of ROS can be toxic to cells; thus, detoxification is essential to the survival of species. In this context, thioredoxin peroxidase (TPx) is a ubiquitous antioxidant enzyme that scavenges peroxides, mainly H_2_O_2_, avoiding oxidative damage in inset cells (Arnér and Holmgren, 2000; Radyuk et al., 2001; Imam et al., 2017). Several TPx were reported in hemipterans (Zumaya-Estrada et al., 2018). In hematophagous arthropods, heme metabolism gives rise to iron that catalyzes the conversion of H_2_O_2_ to hydroxyl free-radical ions, which attack cellular membranes, proteins and DNA (Imam et al., 2017). Thus, TPx could perform a fundamental detoxifying role preventing excessive H_2_O_2_ generation and iron-driven oxygen toxicity. Nevertheless, it is worth pointing that anopheline salivary peroxidases destroy catecholamine and serotonin, thus inhibiting vasoconstrictors that could interfere with insect feeding (Ribeiro and Nussenzveig, 1993; Ribeiro and Valenzuela, 1999). It is possible that TPx may play a multifunctional physiological role.

A member of apolipophorin (ApoLp) family was revealed in Rp 2 sialocomplex. It is an insect hemolymph protein involved in lipid transport. Three ApoLp have been described, ApoLp-I, ApoLp-II, and ApoLp-III (Blacklock and Ryan, 1994), the latter is homologous to the mammalian Apolipoprotein E (Cole et al., 1987) and facilitates the transport of diacylglycerol from the fat body to muscles (Feingold et al., 1995). ApoLp-III was reported to mediate humoral and cellular immune responses (Kim et al., 2004; Whitten et al., 2004; Song et al., 2008; Zdybicka-Barabas and Cytryńska, 2011). It acts as a pathogen recognition receptor (PRR) as it is able to bind to lipoteichoic acid (LTA), present in cell wall of Gram-positive bacteria (Halwani and Dunphy, 1999; Halwani et al., 2001), lipopolysaccharide (LPS) of Gram-negative bacteria (Kato et al., 1994; Halwani and Dunphy, 1997), and beta 1, 3 glucan of fungal cell wall (Whitten et al., 2004). Moreover, ApoLp-III stimulates encapsulation of foreign substances that are too large to be phagocytized by hemocytes in the hemolymph (Zdybicka-Barabas and Cytryńska, 2013).

Heat-shock proteins (HSPs), also known as chaperones, are responsible to tightly control the *de novo* folding, unfolding, assembly/disassembly, translocation, activation/inactivation, disaggregation, and degradation of proteins and protein complexes. HSPs are highly conserved across species and may act through transient interactions, mediating changes in the composition of a protein complex (Makhnevych and Houry, 2012; Finka et al., 2016). HSP70 proteins bind promiscuously to a wide variety of newly synthesized or unfolded proteins, also preventing their aggregation and allowing correct folding (Finka et al., 2016). A HSP70 from *R. prolixus* was reported to be upregulated during the first hours after blood feeding, and knockdown insects showed impaired blood processing and digestion (Paim et al., 2016). In tick saliva, secreted HSP70 may assist the proper folding of proteins involved in the degradation of fibrinogen at the bite site, thus showing a role in fibrinogenolysis during blood feeding (Vora et al., 2017). Here, HSP70 containing oligomers were observed in sialocomplexomes of *T. dimidiata, D. maxima, R. prolixus*, and *R. neglectus*. It is apparent that the folding of triatomine salivary proteins is under the control of this protein, which assists the stability and functionality of proteins (Vora et al., 2017). Furthermore, it has been shown HSP70 assembles in dimers stabilized by post-translational modifications and is maintained in the client-loading complex (Morgner et al., 2015). Future studies on triatomine salivary HSP70 may indicate their specific contribution in the blood feeding context.

Vitellogenin is a yolk precursor lipoprotein secreted in the hemolymph that plays a critical role in oocyte development, as it is a source of nutrients during early stages of development (Smolenaars et al., 2007). As it is involved in lipid transport protein, distinct physiological roles are possible for this protein. Regarding blood-feeding it may enable the uptake and transport of dietary lipids. The vitellogenin lipoprotein was previously recognized in assembly stable homodimers (Smolenaars et al., 2007). Vitellogenin protein was identified in Ti 4 and Rn 2 sialocomplexes.

The identification of protein members that may act in the primary effector mechanisms of immunity in the triatomine sialocomplexomes was expected. Studies on how insect immune molecules interact with self-proteins are scarce. It is likely that a dynamic net of protein-protein interactions may improve the immune response, regulating the molecular events to recognize a variety of foreign targets more efficiently.

In this study, the proteomic analysis revealed a heterogeneous composition in Triatominae sialocomplexomes. From the 72 identifications obtained, 53 were unique, most belonging to the Housekeeping or Enzyme classes. Comparing *R. prolixus* and *R. neglectus* sialocomplexomes, although the identification in the different complexes was not exactly the same, the set of proteins from Secreted class was very similar between them. Concerning the five species, the reduced overlap in proteins composition could be due to changes in gene expression, especially those influenced by adaptive traits. A comparative proteomic analysis of the saliva from *R. prolixus, Triatoma lecticularia*, and *Panstrongylus herreri* showed high interspecific functional biodiversity with only one protein shared among them (Montandon et al., 2016). Moreover, the variation in the salivary protein expression may occur even among individuals within the same species of hematophagous arthropod (Perner et al., 2018). The composition of saliva also changes with time. For instance, in *T. infestans*, recovery of apyrases to maximal activity level takes days after blood feeding, thus suggesting de novo protein synthesis (Faudry et al., 2004b). In addition, even though prior transcriptomic and proteomic analyses of the salivary glands from triatomines have been revealing a conserved set of mRNA that are ubiquitously expressed, they have also found variation in the expression and composition of saliva from triatomines (Santiago et al., 2020). Many of the Secreted proteins have redundant biological roles, and low expression or absence of a protein may not result in impairment of feeding.

The overlap in the protein composition was remarkable among Hypothetically secreted proteins class, indicating that those may be real components of the sialocomplexes. Actin, thioredoxin peroxidase, triabin and uncharacterized (T1HZ69) are shared among the five species, although their positions on complexes showed different range of sizes and low correlated co-migration. It is possible the large salivary complexes occur due to stable homo-oligomeric formations among these proteins, since most are well known to make part of macromolecular complexes in biological systems. It may be worth considering the possibility that smaller secreted proteins such as triabin, antigen-5, nitrophorin, procalin and lipocalin bind with these large macromolecular complexes in a transiently manner, forming multifunctional modules in response to determined stimulus.

All together these results challenge data interpretation but provide significant prediction about native and molecular organization of Triatominae sialocomplexes, suggesting that proteins may undergo several posttranslational modifications to better support stability and functionality not only by one general mechanism, but different routes for oligomerization may occur. Thus, the members of the potential complexes are arranged to form homo- and/or hetero-oligomeric, stable and/or transient assemblies. This dynamic may occur according to the physiological demands of triatomines to give rise to a cooperative functional mechanism, improving the counteraction of host's responses. Finally, the knowledge about the conserved salivary components among triatomine species is fundamental, once may lead to the identification of salivary molecules that might enhance pathogen transmission (Mesquita et al., 2008); and of potential candidates for anti-triatomine vaccines or immunobiologics, concerning their antigenicity and anti-hemostatic and immunomodulatory properties.

## Conclusion

Protein interaction data is sparse in hematophagous arthropods saliva. This is the first report to give an insight into the potential protein complexes present in the saliva of *T. infestans, T. dimidiata, D. maxima, R. prolixus*, and *R. neglectus* kissing bugs by coupling BN-PAGE to LC-MS/MS. Triatominae salivary proteins may form transient homo- and/or hetero-oligomeric complexes arranged in multifunctional modules to efficiently counteract host's hemostasis, act against pathogens acquired during blood feeding, and in the digestion process once swallowed with the host blood. Oligomeric apyrases were identified only in *T. infestans* through proteomic analysis, despite apyrase in-gel activity was observed across all five species. In addition, unexpected secreted proteins were identified composing the Triatominae sialocomplexomes, ascribing putative novel functions to the poorly characterized protein complexes. For instance, inhibition of platelet aggregation and bacterial growth by actin 5C, and degradation of fibrinogen by HSP70. These proteins, which have primarily functions not related to hematophagy in other tissues, may impact the feeding behavior of triatomines. In the future, more efforts will be needed to elucidate protein-specific interactions and their mechanisms of action. Some questions that remain unanswered are: What triggers these transient interactions? What is the relevance of these complexes in blood feeding behavior, vector-host interaction, and vector immunity?

## Data Availability Statement

The datasets presented in this study can be found in online repositories. The names of the repository/repositories and accession number(s) can be found in the article/Supplementary Material.

## Author Contributions

PS, SC, and CA conceived and designed the study. PS and KB were responsible for the triatomines rearing, saliva collection, 1D-BN-PAGE, and zymograms. SM performed LC-MS/MS. PS, CA, SC, and JS performed the data analysis. PS and CA wrote the manuscript. IB, MS, CR, and JS critically revised the manuscript. All authors edited and approved the final manuscript.

## Conflict of Interest

The authors declare that the research was conducted in the absence of any commercial or financial relationships that could be construed as a potential conflict of interest.

## Supplementary Material

Table S1 All saliva protein groups identified from the different bands of the saliva of *Rhodnius prolixus, Rhodnius neglectus, Dipetalogaster maxima, Triatoma dimidiata*, and *Triatoma infestans*, corresponding to the Figure 1.

Table S2 All peptides from the different bands of the saliva of *Rhodnius prolixus, Rhodnius neglectus, Dipetalogaster maxima, Triatoma dimidiata*, and *Triatoma infestans* allowing the protein identification of the sialocomplexes.
